# Reversal of Abnormal CD4+ T Cell Metabolism Alleviates Thyroiditis by Deactivating the mTOR/HIF1a/Glycolysis Pathway

**DOI:** 10.3389/fendo.2021.659738

**Published:** 2021-06-04

**Authors:** Lei Zhao, Qiong Wu, Xiaoli Wang, Shiqi Wang, Xiaoguang Shi, Zhongyan Shan, Weiping Teng

**Affiliations:** ^1^Department of Endocrinology and Metabolism, The Endocrine Institute and The Liaoning Provincial Key Laboratory of Endocrine Diseases, NHC Key Laboratory of Diagnosis and Treatment of Thyroid Diseases, The First Hospital of China Medical University, Shenyang, China; ^2^Department of Pediatrics, Shengjing Hospital of China Medical University, Shenyang, China

**Keywords:** Hashimoto’s thyroiditis, Tregs, HIF1a, immunometabolism, glycolysis

## Abstract

**Background:**

Hashimoto’s thyroiditis (HT) is an autoimmune disease that features activation of thyroid antigen-specific helper T cells. HT patients have increased Th1 and Th17 T cell subsets. Glycolysis supports chronic activation of Th1 and Th17 T cells, but how this contributes to HT remains unknown.

**Methods:**

The metabolism of CD4+ T cells from 30 HT patients and 30 healthy controls was evaluated by determining the extracellular acidification rate (ECAR) and the oxygen consumption rate (OCR). Mice in a subacute thyroiditis (SAT) model were treated with 2DG, metformin, or combination. Metrics of mTOR/HIF-1α/HK2/glycolysis were measured by western blot and Seahorse assay methods. The severity of SAT was measured by flow cytometry and HE staining.

**Results:**

CD4+ T cells from HT patients had enhanced ECAR and OCR. Levels of Glut1, HK2, PKM2, and LDHA in cultured HT CD4+ T cells were elevated. The expression of HK2 and PKM2 in cultured SAT CD4+ T cells was elevated compared with the control group. Activation of the mTOR and HIF-1α pathways was significant in SAT mice, and expression of HIF-1α in the 2DG treated group was reduced. Treatment with 2DG and/or metformin significantly decreased the ratio of Th17 and Th1 T cells.

**Conclusions:**

Thyroiditis results in elevation of the mTOR/HIF-1α/HK2/glycolysis pathway in CD4+ T cells. The activation of this pathway is reduced by treatment with 2DG and metformin, which also reverted imbalances in CD4+ T cell differentiation.

## Introduction

Hashimoto’s thyroiditis (HT) is an organ-specific immune disease characterized by autoantibodies in circulation. HT induces chronic thyroid inflammation and infiltration of thyroid tissue by lymphocytes, including CD4+ and CD8+ T cells (1, 2). In HT, the antithyroid immune response begins with activation of thyroid antigen-specific helper T cells (3). CD4+ T cells undergo activation, proliferation and differentiation after antigen exposure. Depending on different cytokine signals, CD4+ T cells differentiate into different T cell subtypes, including effector T cell (Teff) or T helper cell (Th) lineages, such as Th1, Th2 and Th17 cells, or regulatory T cells (Tregs). Once Th1 and Th17 cells are activated, they induce B cells to secrete thyroid antibodies, contributing to the autoimmune response. Furthermore, Tregs cells suppress immunity and inflammation (4). Study shows that HT patients have elevated Th1 and Th17 subsets and exhibit excessive expression of IFN-γ and IL-17 (5). Therefore, targeting the pro-inflammatory T helper subtypes, such as Th1 and Th17 cells, could be a promising treatment strategy for HT.

In order to undergo proliferation and differentiation, activated CD4+ T cells must use a metabolic pathway that meets energy requirements necessary to support rapid biosynthesis. In contrast with the TCA cycle, glycolytic metabolism provides less ATP but more key metabolic intermediates that can benefit cellular growth (6). T cells activated by lipopolysaccharide (LPS) preferentially use the glycolysis metabolism pathway. Activated T cells show an increase in glycolysis, most notably in Th17 cells and Th1 cells (7, 8). While the metabolic characteristics of CD4+T cells in LPS-induced inflammation have been described, the metabolism of CD4+ T cells remains poorly understood in the context of HT.

HIF-1α is a key metabolic sensor in cellular metabolism pathways, and plays a vital role in influencing immune responses (9, 10). The switch from oxidative phosphorylation to glycolysis during immune cell activation requires HIF-1α (11). HIF-1α protein translation is enhanced by activation of the PI3K/AKt/mTOR pathway (12). Different T cell subsets apply different metabolic pathways depending on their specific cellular demands (13). T cell fate can be shaped by differential metabolic programming (14). We hypothesize that cellular metabolism contributes to HT pathogenesis through T cell activation, that some metabolic sensors, such as mTOR and HIF-1α, influence autoimmune responses through metabolic pathways in HT, and that metabolic modulating treatments could be used to reduce or revert HT.

In the present study, we used in a murine model of spontaneous autoimmune thyroiditis (SAT) induced by excessive iodine intake in NOD-2^h4^ mice. We evaluated whether treatment with the glucose blocker 2-deoxyD-glucose (2DG) and/or the mitochondrial complex 1 inhibitor, metformin, could alter the relationship between abnormally activated mTOR/HIF-1α/glycolysis and imbalance of CD4+ T cells subtypes. The results of this study provide new insights into how metabolism regulates T cell function and autoimmunity, and reveal key modulators of metabolism in the pathology of autoimmune thyroiditis.

## Methods

### Human Subjects

The HT group consisted of 30 HT patients (4 males, 26 females, 35–65 years old) (**Table 1**). A clinical diagnosis of HT was based on elevated serum levels of thyroid antibodies (TPOAb> 200 IU/ml). None of the HT patients had accompanying thyroid carcinoma, and none had a medical history of other autoimmune diseases. The healthy control (HC) group consisted of 30 participants (7 males, 23 females; 35–65 years). The healthy control group comprised of age- and sex-matched healthy individuals with no history of autoimmune disease and with normal rage of thyroid antibody and thyroxin levels. None of the participants had taken any medicine. The study protocol was approved by the Ethics Committee of China Medical University (Liaoning, China).

**Table 1:** Characteristics of patients with HT and healthy controls in this study.

	Healthy Controls (n=30)	HT Patients (n=30)	P-value
**Age(years)**	39.93±12.19	47.13±2.85	0.218
**Gender(female)**	23(77%)	26(87%)	0.000
**TSH (mIU/L)**	2.06±0.16	2.85±0.28	0.004
**FT4 (pmol/l)**	15.13±0.39	14.50±0.31	0.228
**FT3 (pmol/l)**	4.9±0.11	4.6±0.16	0.014
**TPOAb (IU/ml)**	12.70±4.21	422.59±26.36	0.000
**TgAb (IU/ml)**	97.24±39.54	368.35±65.03	0.027

### Animal Subjects and SAT Models

NOD.H-2h4 mice were purchased from the Jackson Laboratory (Bar Harbor, ME, USA). Mice were breed and raised in specific pathogen-free conditions with a 12 h light/12 h dark cycle at the Animal Experiment Center of China Medical University. All animal experiments were approved by the Animal Ethics Committee of China Medical University. Seven mice were selected and randomized into an iodine-free water control group and 28 mice were assigned into a high-iodine group. The iodine-free group received sterile water for 12 weeks. After 4 weeks of sterile water, the high-iodine group was administered 0.05% NaI (1000 x higher than normal concentration) for 8 weeks, then switched back to sterile water for 4 weeks. Treatment was performed for 4 weeks with 3 mg/mL metformin (Met; Sigma) or 5 mg/mL 2DG (Sigma), or a combination of the two (n = 7 mice per treatment group), administered in drinking water. All the mice were euthanized after 12 weeks. Thyroid sections were collected and prepared for hematoxylin and eosin staining. The expansion, lesions, and lymphocyte infiltration of thyroid follicles were assessed for indications of spontaneous autoimmune thyroiditis (SAT). The SAT condition was identified based on the collective area of the inflammatory lymphocyte cells, as previously described (15): 0, few inflammatory cells and mild abnormal follicular cells; 1+: <10% infiltration; 2+: 10–30% infiltration; 3+: 30–50% infiltration; 4+: more than 50% infiltration. Histological scores of thyroiditis were independently estimated by two blinded scorers.

### Flow Cytometric Analysis of T-Cell Subsets

Lymphocytes were sterilely prepared from harvested spleens or blood of participants, stimulated by CD3/CD28 (Dynabeads Human/mouse T-Activator, Life Technologies), and incubated at 37°C for 5 h. Anti-mouse/human CD4 and CD25 antibody staining (BD, USA) was used to detect extracellular proteins. Anti-mouse/human IFN-γ, IL-4, and IL-17a antibody staining (BD, USA) was used to detect intracellular proteins. The proportions of T-cell subsets were measured and analyzed using FACScan Flow Cytometry and WinMDI2.9.

### Metabolic Measurements

Human CD4+ T cells were enriched from peripheral blood using the RosetteSep^®^ Enrichment Cocktail (StemCell Technologies). Cells were cultured with CD3/CD28 (Dynabeads Human T-Activator, Life Technologies) for 3 days with 20 U/mL IL-2. lymphocytes were sterilely prepared from harvested spleens, stimulated by CD3/CD28 (Dynabeads Human/mouse T-Activator, Life Technologies), and incubated at 37°C for 3 days. 1*10^6^ CD4+ T cells were cultured in XFe96 plates coated with Cell-Tak. The basic assay media contains 2.5 uM dextrose and 2 mM glutamine. After cultured for 2 hours, injections of 2 μM oligmycin, 2 μM FCCP, 10 mM 2-deoxyglucose, and 0.5μM rotenone/antimycin A were performed sequentially. ECAR and OCR were measured using a XF96 Extracellular Flux Analyzers under mitochondrial stress test conditions (Seahorse, Agilent Cell Analysis Technology, USA) and glycolysis stress test conditions (Seahorse, Agilent Cell Analysis Technology, USA). The Agilent seahorse XF technology can measure changes in the rate of extracellular acidification (ECAR, a qualitative indicator of lactate in glycolysis) and oxygen consumption rate (OCR). Basal OCR or ECAR represents the last measurement before first injection. Stressed OCR or ECAR represents maximum rate measurement after oligomycin (ECAR)/FCCP (OCR) injection. The metabolic potential represents (stressed/basal) *100%.

### Western Blotting

Total cellular proteins were extracted from CD4+T cells and spleen mononuclear cells using a protein extraction kit (Keygen Biotech, Nanjing, China). Proteins were separated by 10% SDS/PAGE and transferred to PVDF membranes (Merck Millipore, USA). Membranes were washed three times with TBST, then blocked in TBST containing 5% BSA for 2 h at room temperature. Membranes were then incubated overnight at 4°C with anti-Sirt2, anti-HIF1a, anti-mTOR, anti-LDHA, anti-Glut1, anti-HK2, anti-PKM2 antibody (1:1000 dilution; Cell Signaling Technology) and anti-GAPDH(1:1000; Santa Cruz). Membranes were washed in TBST three times, and then incubated with goat anti-Rabbit IgG secondary antibody (1:5000 dilution; Santa Cruz) solution for 2 h at room temperature. Finally, protein bands were visualized using a chemiluminescence western blot detection system (Alpha Innotech; MicroChemi 4.2).

### Statistical Analysis

Statistical analyses were performed using SPSS 17.0 software and GraphPad Prism 6.0 software. Sample mean values were compared using independent sample t-tests with a level of significance at P < 0.05. Proportion value comparisons were performed using the χ2 - test. Each *in vitro* experiment was performed at least twice with reproducible results.

## Results

### Subtypes of CD4+ T Cells Are Imbalanced in HT Patients

**Figure 1:**
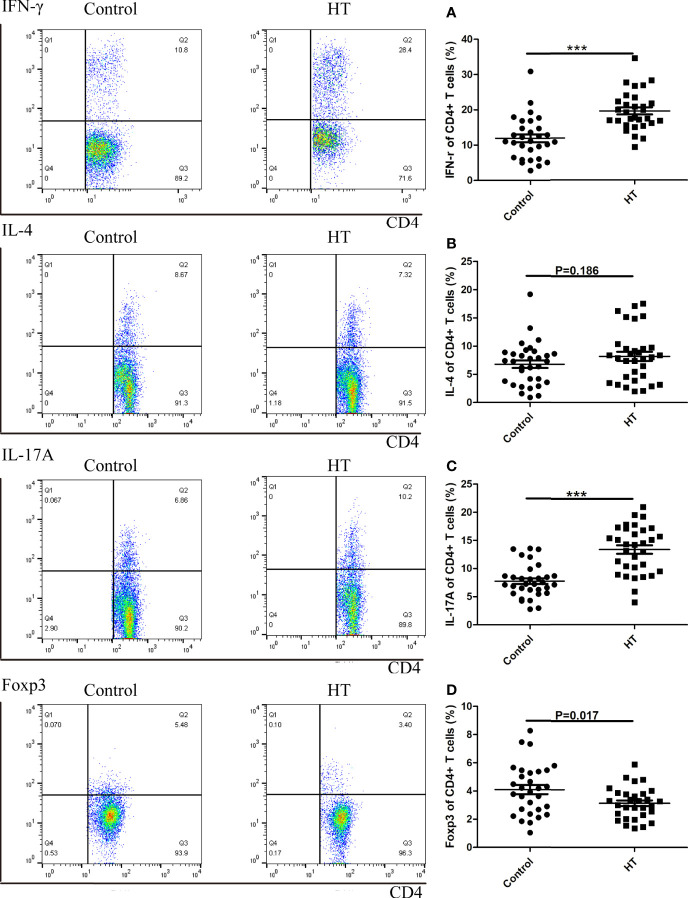
The ratio of CD4+ T cells subtypes in HT patients and HC group determined by flow cytometry. A representative Flow sample is shown from each sample, and samples were run individually rather than pooled. Lymphocytes from HC and HT subjects were stimulated for 5 hours with CD3/CD28 and the ratio of subtypes in CD4+ T cells were measured by flow cytometry. The cell frequency of CD4+IFNγ+ **(A)**, CD4+IL4+ **(B)**, CD4+IL17A **(C)** and CD4+Foxp3+ **(D)** was determined. The frequency of T cell subsets was investigated in sixty donors (30 healthy and 30 HT donors). The mean cytokine values are compared using two-way ANOVA and the p values are indicated in the figure. (***P < 0.01).

We analyzed the percentage of CD4+ IL17A+, CD4+ IFN-r+, CD4+ IL4+ T cells and CD4+ CD25+ Tregs in the total number of CD4+ T cells from peripheral blood of 30 patients with HT and 30 healthy control participants (HC) by flow cytometry. In the HT patients, the ratio of the CD4+ IL17A+(7.49% vs. 2.72%, p<0.01) and CD4+ IFN-r+ T cells (14.23% vs. 8.85%, p<0.01) was significantly higher than that in the HC group (**Figures 1A, C**). Additionally, the ratio of CD4+ Foxp3+ Tregs (3.50% vs. 7.54%, p<0.01) was significantly lower than in the HC group (**Figure 1D**). However, there was no difference between two groups in the ratio of CD4+ IL4+ T cells (1.28% vs. 0.97%, p=0.168) (**Figures 1B**).

### Abnormal Cellular Metabolism in CD4+ T Cells of Patients With Hashimoto’s Thyroiditis

**Figure 2:**
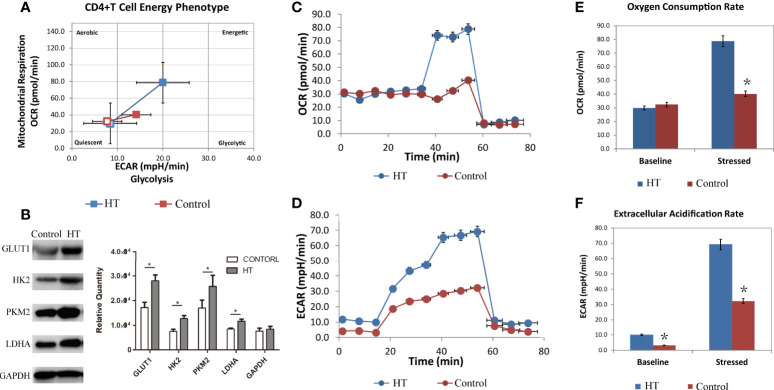
The metabolism characteristic of CD4+ T cells from HT patients and HC group. The overall phenotype of CD4+ T cells **(A)** of HT patients (blue) and HC group (red). The hollow squares represent the basal ECAR (X-coordinate) and OCR (Y-coordinate), and the solid squares represent the stressed ECAR (X-coordinate) and OCR (Y-coordinate). Both basal OCR and stressed OCR of CD4+ T cells **(C, E)** and basal ECAR and stressed ECAR **(D, F)** in two groups were measured by seahorse after incubated with CD3/CD28 for 3 days. The expression of glycolysis related key enzyme (Glut1, HK2, PKM2 and LDHA) in cultured HT CD4+ T cells **(B)** were measured by western blot. ECAR, extracellular acidification rate. OCR, oxygen consumption rate. *P < 0.05.

To test whether these CD4+ T cell subtypes exhibited alterations in cellular metabolism, we evaluated extracellular acidification rate (ECAR), which is primarily indicative of glycolysis, and the oxygen consumption rate (OCR), which corresponds to aerobic metabolism. CD4+ T cells from HT patients showed enhanced ECAR and OCR compared to CD4+ T cells from the age-matched healthy control group (**Figure 2A**). The difference in CD4+ T cell OCR is not apparent at basal metabolism levels but became more pronounced in stressed conditions (**Figures 2C, E**). However, the basal ECAR of CD4+ T cells in HT patients was higher than the HC group (**Figure 2D**). This indicates that the basal state of CD4+T cells from the two groups are different. Stressed CD4+ T cells from the HT group also exhibited higher ECAR (**Figure 2F**). Elevated ECAR and OCR in the stressed cells indicated an increased capacity for glycolysis and aerobic respiration in the CD4+ T cells from the HT group. These results suggest that the increased metabolism of CD4+ T cells in HT patients is required to support the activated immune functions.

In order to further evaluate glycolysis in these cells, we extracted protein from CD4+T cells to analyze the expression of key glycolysis enzymes. Western blot results show that the expression of Glut1, HK2, PKM2, and LDHA was elevated in cultured CD4+ T cells from the HT group compared with the HC group (**Figure 2B**). This result suggests that the increased metabolism of CD4+ T cells in HT patients results from increased expression of glycolysis-associated enzymes. Overall, CD4+ T cells from HT patients present with enhanced cellular metabolism that may contribute to the imbalance in ratios of CD4+ T cell subtypes.

### Reprogramming Abnormal Metabolism of CD4+ T Cells in SAT Mice by Administration of Metabolic Regulatory Agents

We addressed whether pharmacological intervention against abnormal metabolism using metabolic regulatory agents (2DG, metformin, combination) was effective in a murine model of SAT induced by excess dietary iodine in NOD-2^h4^ mouse. After 8 weeks, twenty-one mice were randomized into three groups for treatment with 2DG, metformin, or a combination of 2DG and metformin (n = 7 per group).

**Figure 3:**
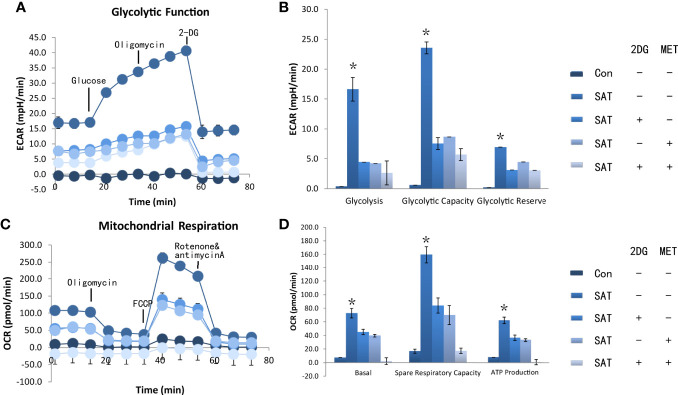
2DG and metformin reverted CD4+ T cell metabolic dysfunction *in vivo*. The NOD-2h4 mice were raised and given excess iodine to form a murine model of SAT. After 8 weeks three groups of mice for treatment with 2DG, metformin, or a combination of 2DG and metformin (n = 7 per group). CD4+ T cells was extracted from these mice and the basal ECAR **(A)**, glycolysis capacity and glycolysis reserve of CD4+ T cells of ever group **(B)**. The basal OCR, spare respiration capacity and ATP production of CD4+ T cells of every group were measured by seahorse **(C, D)**. ECAR, extracellular acidification rate; OCR, oxygen consumption rate. *P < 0.05.

**Figure 4:**
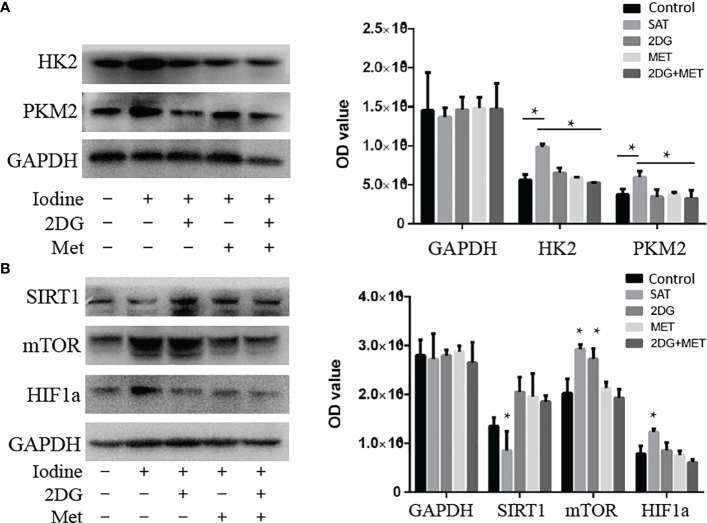
Treatment with 2DG and/or metformin deactivated the mTOR/HIF-1α/HK2/PKM2 pathway in CD4+ T cells of SAT mice. CD4+ T cells were extracted from SAT mice treated with 2DG, metformin, or combination, and expression of glycolysis-related enzymes were evaluated by western blot (n = 7 per group). **(A)** The expression of key glycolysis-related enzymes (HK2 and PKM2) of cultured SAT CD4+ T cells were measured by western blot. **(B)** The expression of key pathway related proteins (sirt1, mTOR, and HIF-1α) were measured by western blot.

We found that 2DG inhibited CD4+ T cell ECAR in SAT mice (**Figure 3A**), and decreased OCR (**Figure 3C**), likely by decreasing glucose oxidation. Metformin also decreased ECAR (**Figure 3A**) and OCR (**Figure 3C**). The combination of the two regulators showed the same trend in reducing ECAR (**Figure 3B**), and had a combinatorial effect in reducing OCR and ATP production (**Figure 3D**). Western blot analysis showed that the expression of HK2 and PKM2 in cultured SAT CD4+ T cells was elevated compared with the control group, and revealed that treatment with 2DG and metformin significantly reduced the elevation of glycolysis-related enzymes in CD4+ T cells from SAT mice (**Figure 4A**).

In order to understand the abnormal pathways and to identify key factors contributing to this abnormal metabolism, we analyzed levels of mTOR/HIF-1α and Sirt1 in the CD4+T cells of SAT mice. The expression of Sirt1 was reduced in SAT mice and went back to the normal expression following treatment with 2DG and/or metformin (**Figure 4B**). The mTOR and HIF-1α levels in SAT mice were all elevated. Treatment with 2DG only reduced HIF-1α expression, and the effects on mTOR levels were more significant in the metformin treatment group (**Figure 4B**). Overall, the mTOR/HIF-1α/HK2/glycolysis pathway of CD4+ T cells in SAT mice was activated, and treatment with the metabolic regulators 2DG and metformin could reprogram this abnormal activation.

### Treatment With 2DG and Metformin Alleviates Thyroiditis Induced by Excess Iodine in NOD-2h4 Mice

**Figure 5:**
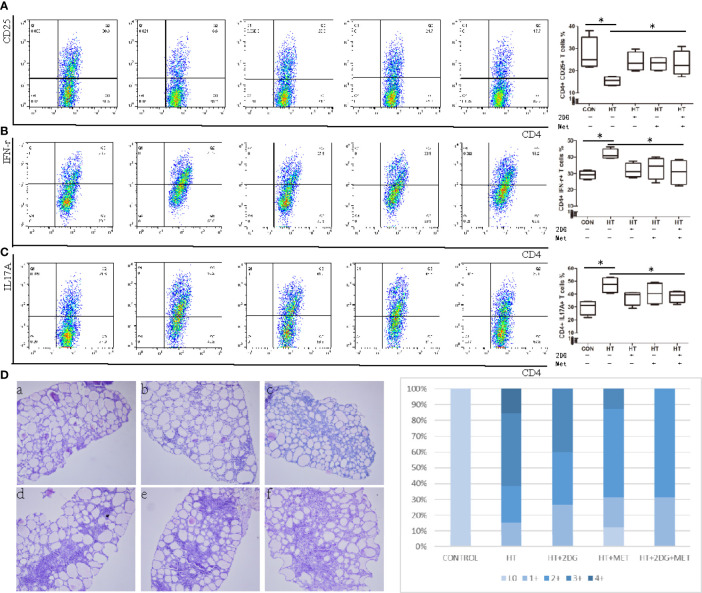
Treatment with 2DG and/or metformin for 4 weeks reversed thyroiditis in SAT mice. SAT mice treated with 2DG, metformin, or combination, and spleen mononuclear cells were harvested, incubated, and evaluated by flow cytometry (n = 7 per group). Spleen mononuclear cells were incubated and the subtype of CD4+ T cells including CD4+CD25+ Tregs T cells **(A)**, CD4+IFNγ+ Th1 T cells **(B)**, CD4+CD17A+ Th17 T cells **(C)** were measured by flow cytometry. The severity of SAT, indicated by proportions of high and low severity scores, was evaluated **(D)**. Severity score: 0(a), 1+(b), 2+(c), 3+(d), 4+ (e, f). *p < 0.05.

We sought to determine if metabolic reprogramming with 2DG and metformin could reduce indications of SAT in mice. Treatment with 2DG and/or metformin *in vivo* up-regulated Treg T cells (**Figure 5A**), and significantly decreased the elevated ratio of Th1 (**Figure 5B**) and Th17 (**Figure 5C**) cells in SAT mice. The treatments significantly decreased the severity of SAT, indicated by a lower proportion of mice with high severity scores in the treatment groups (**Figure 5D**). However, the combination of 2DG and metformin did not have an added effect in reducing indications of SAT over individual treatments. Overall, treatment with 2DG and/or metformin could reverse the imbalance of CD4+ T cells in SAT mice and reduced inflammation in thyroid tissue.

## Discussion

Hashimoto’s thyroiditis (HT), discovered by Hakaru Hashimoto in 1912, is an organ-specific autoimmune disease (16). As one of the most widespread thyroid disorders that can lead to hypothyroidism (17, 18), HT is characterized by lymphocytic infiltration in thyroid tissue, follicular cell destruction, and the presence of thyroid-specific autoimmune antibodies. Environmental factors can trigger thyroid autoimmunity in individuals susceptible to HT, resulting in increased thyroid antigen presentation and reduced self-tolerance in the thyroid (19). Consequently, pro-inflammatory cytokines are produced by immune cells and thyroid cells, resulting in elevated Th1 and Th17 responses (20). These proinflammatory subtypes of immune cells produce factors that lead to thyrocyte apoptosis and thyroid destruction (19). Regulatory T cells are vital for maintaining self-tolerance and reducing excessive immune responses. The reduction in or functional impairment of Tregs also influences the pathogenesis of HT (21). In this study, we demonstrate that patients with HT exhibit increased ratios of Th1 and Th17 T cell subtypes, and decreased ratios of Tregs.

Recently, the idea of “immunometabolism” has risen in prominence, and refers to how immune cells can switch to preferential metabolic pathway to generate energy carriers and metabolic intermediates when they need to produce biomass and inflammatory mediators. Indeed, the different functional subtypes of helper T cells preferentially utilize different metabolic pathways (8, 22). Not surprisingly, the factors that influence metabolism, including key enzymes in metabolic pathways, affect the differentiation of naïve T cells. Therefore, we analyzed the metabolic characteristics of CD4+ T cells in patients with HT. Compared to a healthy control group, the CD4+ T cells of HT patients has basal metabolism indicating high glycolysis and normal aerobic metabolism. However, the stressed ECAR and OCR levels were both higher in CD4+ T cells form HT patients than from the healthy control group. This suggested that CD4+T cells switch to high glycolysis and slightly high aerobic metabolism in order to maintain immune activation. This result agreed with the changes in immunometabolism described in systemic lupus erythematosus (SLE) (23). However, the alterations in immunemetabolism in rheumatoid arthritis (RA), another autoimmune disease, were much different. In RA, glycolysis of CD4 T cells was very low, and the pentose phosphate pathway (PPP) was elevated (24).

The activation of CD4+ T cells increases both lactate production and oxidative phosphorylation (25, 26). Additionally, differentiation into inflammatory CD4+ T cell subtypes, especially Th1 and Th17 cells, relies on aerobic glycolysis (27, 28). CD4+ T cell activation requires mitochondrial metabolism, specifically the production of reactive oxygen species (29). In our study, we showed that elevated glycolysis results from activation of key glycolysis-related enzymes in CD4 T cells, including Glut1, HK2, PKM2, and LDHA. Based on these observations, we administered metabolism regulatory agents, 2DG and metformin, in a model of SAT induced by excessive iodine intake in NOD-2^h4^ mice. In a model of SLE, 2DG and metformin significantly delayed disease onset (30). Our study showed that treatment of SAT mice with 2DG and/or metformin reverted the elevated glucose metabolism and mitochondrial oxidation of CD4+ T cells. 2DG and metformin efficiently normalized chronically activated CD4+ T cells and inhibited both aerobic glycolysis and oxidative phosphorylation. Our *in vivo* studies specifically compared pyruvate oxidation and reduction in CD4+ T cell activation and polarization into Th1 and Th17 subsets.

Sirtuin 1 (SIRT1), a member of the HDAC sirtuin family, is a crucial factor in metabolism and immune responses (31, 32). SIRT1 can deacetylate downstream targets, including hypoxia inducible transcription factor-1 alpha subunit (HIF-1α) (33), which is a key transcriptional factor in pro-inflammatory responses and cellular metabolism. Recent studies have shown that HIF-1α is responsible for glycolytic responses downstream of mTOR, however SIRT1 exerts negative regulatory effects on mTOR (34). Changes in HIF1a expression lead to altered differentiation of naïve T cells (35). Moreover, abnormal expression of SIRT1 has been associated with elevated production of thyroid autoantibodies (36). In our study, chronically activated CD4+ T cells had lower expression of SIRT1 and elevated levels of mTOR and HIF-1α.

In summary, we identified abnormal CD4+ T cell metabolism as a therapeutic target in a murine model of SAT. 2DG and/or metformin may also directly target immune cells *in vivo*. Glycolysis is essential for Th1 and Th17 cell functions, and both glycolysis and mitochondrial function are vital for the activation of CD4+ T cells. In HT and a SAT model, the activation of glycolysis in CD4+ T cells resulted from activation of the mTOR/HIF-1α pathway. Although 2DG and metformin proved effective at reversing the disease phenotype *in vivo*, and whether these therapies could be applied to treat HT in human patients remains unknown. This study only spotted out the overall metabolism changes in the CD4+T cells in HT patients. However, due to the limited volume of patients’ peripheral blood we could collected, the different metabolism changes in different subtypes of CD4+ T cells could not be measured. Hence, we could not explain the phenomenon in this study which is glycolysis and aerobic oxidation enhanced at the same time. Finally, we demonstrate that deactivation of SIRT1 could be related to regulation of the whole metabolic pathway in CD4+ T cells, but further study is required to evaluate this phenomenon.

## Data Availability Statement

The raw data supporting the conclusions of this article will be made available by the authors, without undue reservation.

## Ethics Statement

The study protocol was approved by the Ethics Committee of China Medical University (Liaoning, China). The patients/participants provided their written informed consent to participate in this study.

## Author Contributions

XS conceived of and designed the study; ZS and WT organized and supervised the study; LZ and QW conducted the experiments of this study. XW and SW conducted the statistical analysis. LZ and QW drafted the article. All authors contributed to the article and approved the submitted version.

## Funding

This study was funded by National Natural Science Foundation of China (Grant No. 81870543) and Research Foundation,Department of Science and Technology, Liaoning Province Government,China (Grant no. 20180530060).

## Conflict of Interest

The authors declare that the research was conducted in the absence of any commercial or financial relationships that could be construed as a potential conflict of interest.
